# The Effectiveness of Osseodensification Drilling Protocol for Implant Site Osteotomy: A Systematic Review of the Literature and Meta-Analysis

**DOI:** 10.3390/ma14051147

**Published:** 2021-02-28

**Authors:** Alessio Danilo Inchingolo, Angelo Michele Inchingolo, Ioana Roxana Bordea, Edit Xhajanka, Donato Mario Romeo, Mario Romeo, Carlo Maria Felice Zappone, Giuseppina Malcangi, Antonio Scarano, Felice Lorusso, Ciro Gargiulo Isacco, Grazia Marinelli, Maria Contaldo, Andrea Ballini, Francesco Inchingolo, Gianna Dipalma

**Affiliations:** 1 Department of Interdisciplinary Medicine, University of Medicine Aldo Moro, 70124 Bari, Italy; ad.inchingolo@libero.it (A.D.I.); angeloinchingolo@gmail.com (A.M.I.); donatoromeo@gmail.com (D.M.R.); mromeo@myuax.com (M.R.); czapp@myuax.com (C.M.F.Z.); giuseppinamalcangi@libero.it (G.M.); drciroisacco@gmail.com (C.G.I.); graziamarinelli@live.it (G.M.); francesco.inchingolo@uniba.it (F.I.); giannadipalma@tiscali.it (G.D.); 2 Department of Oral Rehabilitation, Faculty of Dentistry, Iuliu Hațieganu University of Medicine and Pharmacy, 400012 Cluj-Napoca, Romania; 3 Department of Dental Prosthesis, University of Tirana, Nr 183 Tirana, Albania; editxhajanka@yahoo.com; 4 Freelancer Studio Dentistico Drs. Romeo, 75025 Policoro, Italy; 5 Department of Medical, Oral and Biotechnological Sciences, University of Chieti-Pescara, 66100 Chieti, Italy; ascarano@unich.it; 6 Human Stem Cells Research Center HSC of Ho Chi Minh, Ho Chi Minh 70000, Vietnam; 7 Embryology and Regenerative Medicine and Immunology, Pham Chau Trinh University of Medicine Hoi An, Hoi An 70000, Vietnam; 8 Multidisciplinary Department of Medical-Surgical and Dental Specialties, University of Campania Luigi Vanvitelli, Via Luigi de Crecchio, 6, 80138 Naples, Italy; maria.contaldo@unicampania.it; 9 Department of Biosciences, Biotechnologies and Biopharmaceutics, Campus Universitario “Ernesto Quagliariello” University of Bari “Aldo Moro”, 70125 Bari, Italy; andrea.ballini@uniba.it; 10 Department of Precision Medicine, University of Campania “Luigi Vanvitelli”, 80138 Naples, Italy

**Keywords:** osseodensification bone osteotomy, endo-osseous dental implant, primary stability, bone to implant contact

## Abstract

Many different osteotomy procedures has been proposed in the literature for dental implant site preparation. The osseodensification is a drilling technique that has been proposed to improve the local bone quality and implant stability in poor density alveolar ridges. This technique determines an expansion of the implant site by increasing the density of the adjacent bone. The aim of the present investigation was to evaluate the effectiveness of the osseodensification technique for implant site preparation through a literature review and meta-analysis. The database electronic research was performed on PubMed (Medline) database for the screening of the scientific papers. A total of 16 articles have been identified suitable for the review and qualitative analysis—11 clinical studies (eight on animals, three on human subjects), four literature reviews, and one case report. The meta-analysis was performed to compare the bone-to-implant contact % (BIC), bone area fraction occupied % (BAFO), and insertion torque of clockwise and counter-clockwise osseodensification procedure in animal studies. The included articles reported a significant increase in the insertion torque of the implants positioned through the osseodensification protocol compared to the conventional drilling technique. Advantages of this new technique are important above all when the patient has a strong missing and/or low quantity of bone tissue. The data collected until the drafting of this paper detect an improvement when the osseodensification has been adopted if compared to the conventional technique. A significant difference in BIC and insertion torque between the clockwise and counter-clockwise osseodensification procedure was reported, with no difference in BAFO measurements between the two approaches. The effectiveness of the present study demonstrated that the osseodensification drilling protocol is a useful technique to obtain increased implant insertion torque and bone to implant contact (BIC) in vivo. Further randomized clinical studies are required to confirm these pieces of evidence in human studies.

## 1. Introduction

In recent years, the osseointegrated dental implant has become the gold standard therapy to avoid missing teeth loss [1,2,3]. The osseointegration is an ankylotic relationship between two interfaces, respectively, the implant surface and the surrounding bone. The healing of dental implant is clinically and histologically determined by the primary stability, that is, the expression of the friction ratio during the screw positioning, while the secondary stability is correlated to the new bone formation and remodeling in contact with the implant surface [4,5].

Today, new techniques have been developed [6] to decrease the tissue stress [7], and hence the pain and some complications to the patient [8], and make the performance of the surgery moment for the dentist and his team easier [9]. In this paper, we analyzed the osseodensification technique operating in the opposite rotatory direction than the conventional drills due to the use of different drills with an exclusive and patented design. Because of this technique, it is possible the bone condensing toward the osteotomy walls within the same surgery moment of the implant site preparation [9,10,11,12,13,14,15].

Nowadays, dental implants have become the treatment adopted for the replacement of natural dental elements [16]; this is due to the high biocompatibility and great biomechanical properties; therefore, these are well accepted by patients who require this treatment more and more frequently [17]. The placement of a dental implant involves one surgery moment, a prosthetic moment, and a step of periodic follow-up to assess the success and the maintaining of the ideal conditions of dental implants and patients’ tissues [18]. There are some factors that may influence the result of the treatment; some depend on the patient, such as the presence of systemic diseases (diabetes mellitus, diseases of coagulation, osteoporosis) [19,20,21,22,23], therapy with anticoagulants, bisphosphonates, cardio aspirin [24], physiology and anatomy of the treated structured (bone quantity available and density, mental nerve not far from the level of the bone crest) [25,26,27,28,29,30,31,32]; others depend on the operator (experience, methods, and instruments used, team skills) [33]. Nevertheless, we must consider that also in healthy patients and experienced operators, some implant complications (peri-implantitis, bone dehiscence, and impossibility to obtain ideal implant stability) may be a very common situation because of other etiologic agents, such as biomechanical factors or inadequate preparation of the site hosting the implant [19,24,25,26,33,34]. Moreover, the bone density evaluation through preoperative tomography planning could be useful for the qualitative and quantitative diagnostic of the native alveolar ridges according to the Hounsfield scale [35]. These values, in conjunction with resonance frequency analysis (RFA) values and insertion torque measurements, can provide the implant surgeon with an objective assessment of bone quality and may be especially useful where a poor-quality bone is suspected.

The evolution of the techniques and materials adopted has allowed more doctors and patients to use this type of therapy, making possible the placement of implant elements in very hard situations where only a few years ago the professional would have chosen a different therapeutic choice [36]. One of the main principles for successful therapy is the achievement of suitable primary stability during the implant placement [37] in respect to the biology of the host [38] and factors depending on the invasiveness of the operation; the more the preparation of the implant site will be performed in an atraumatic way by avoiding the overheating, and so the necrosis of the site, the more we will be able to respect tissues of the host by avoiding intra- and post-operation complications (bleeding, swelling, local infection, invasion of the noble structures adjacent to the surgery, implant early loss, inadequate healing of hard and soft tissues involved during the operation, presence and/or formation of pus immediately after the operation, pain, alteration of the sensitivity of the area) [39,40,41].

After the surgery, we may assess the primary stability of the placed implants, a value that indicates the contact of the implant surface with the surrounding bone [42]; after this, the secondary stability will follow, which is reached after the processes of remodeling and healing of the bone [43]; usually, the achievement of good primary stability will be followed by correct secondary stability [44]. In this way, the dynamic functional response of the bone tissue is determined by the bone-to-implant contact percentage (BIC), which is constantly interested in remodeling processes under the functional loading [25,26,40,41,42,43,45,46,47,48]. In order to assess the implant stability, we may use an index called the implant stability quotient (ISQ), a unit of measurement, which allows us to assess the degree of integration of the placed implants [49]; the clinical range of the ISQ is ranged between 55 and 80, and if the value is higher than 65, it is commonly accepted as a favorable situation for implant stability; on the contrary, values under 45 are considered as insufficient implant stability [42].

The ISQ has no relation with the micromovements suffered by the implants [50], representing another factor to consider fromnthe beginning of the post-operation step because if it is higher than 50–100 µ, it may influence negatively the militainment of the implant stability [51,52]. Moreover, the insertion torque (IT) represents one of the most common clinical predictors for dental implant primary stability [11,14,15,53,54]. This value is correlated to the mechanical frictional relationships between the implant fixture and the surrounding bone during the device positioning. The disadvantage of IT is represented by the non-repeatability of this measurement during the operative practice [11,53].

Therefore, we may consider implantology as the science that has led to a new revolution in the field of oral rehabilitation, with a success rate of more than 90% in the last decade, whose success factors are due to many factors, which we can sum up in [54] as factors related to implants (biocompatibility, the topography of the surface, composition, shape, ergonomics, dimension); factors related to the host (quality, density, the volume of the bone tissue); factors related to the surgery (primary stability obtained, infections, mechanic and/or thermal mechanic trauma); and systemic factors (systemic diseases, administration of drugs, parafunctional habits) [55,56,57,58].

Among the mentioned factors, we chose to focus on the primary stability because this is an indicator of the predictability of healthy that the implant will keep by the time and therefore the success of the therapy [59]. Over the years, several techniques have been developed to increase the primary stability; some of those include the use of condensers of bone tissue and osteotomes, namely, specific tools to increase the bone quantity used as anchorage for the implant [60]. Despite the success of the use of these techniques is supported by the scientific community, they have considerable complications and sometimes they appear to be difficult to perform [61].

The recent technique of osseodensification introduced by Huwais in 2015 allows us to increase the bone tissue density surrounding the preparation implant site during the surgery with adequate drills designed working in opposite direction, with low-speed irrigation (by avoiding the overheating of the tissue, and so its necrosis) [62]. The purpose of this review is to perform an analysis of scientific texts issued until now about this topic and the bone-to-implant contact % (BIC), bone area fraction occupied% (BAFO), and insertion torque meta-analysis evaluation. The aim of the present research was to investigate the osseodensification drilling procedure for implant site osteotomy through a systematic review and meta-analysis. This review has been developed to define the advantages, the eventual complications, the unexpected events, the success rate, and the efficacy of the preparation of the implant site occurred through the use of the innovative technique using proper drills for the osseodensification; to obtain the needed information, we performed a careful quantitative analysis of the modern literature.

## 2. Materials and Methods

### 2.1. Search Strategy

The PICO (population, intervention, comparison, outcome) question has been reported in Table 1. The aim of this article is to analyze the results of modern studies on osseodensification technique and evaluate the cases in which it could be beneficial in comparison to the common technique, the anatomical areas where the technique is more effective because of their peculiar kind of cut, and the capacity of this technique to reach a primary stability value higher than the common methods, especially in difficult cases.

We have performed this research in the archives PubMed–Medline and Google Scholar, without limit of language, written from 2012 to 2020. The following keywords have been researched singly and together with the Boolean operators “or, not, and”: “osseointegration,” “osseodensification,” “drill,” “stability,” “primary,” “implant,” “dental”; 818 papers were founded using these keywords. Subsequently, we selected the most important papers that mostly met the inclusion criteria that we set for the development of this scientific review. Then, these papers have been analyzed to answer the question that has stimulated the production of this text “what are the clinical and histological effects at the level of the bone tissue obtained through the preparation of the implant site with the technique of osseodensification?”. To avoid the risk of bias and to respect PRISMA Statement [63], we only selected the papers that describe the technique of bone compaction with drills specific for this preparation, both used with clockwise and anticlockwise movement, with refrigeration, and with a salt solution. We considered the studies with a statistic value *p* < 0.005, and for the choice of papers concerning operations on animals, we only selected those that followed the guidelines ARRIVE [26]. The pictures included in this paper have been obtained through research in the archive PubMed–Medline, Google Scholar, and clinical cases managed by the authors of this review. The data recorded from the analyzed studies were duplicated in this article from the original ones to avoid manipulation or errors that can happen in the data transcription.

Among the research of the archives of scientific literature obtained by the keywords previously mentioned, according to the impact factor, the relevance of the title and summary, and the year of publication, we have carried out the first step of this selection of those used in this review and then we have chosen the most specific and suitable to the aim of our research.

### 2.2. Inclusion and Exclusion Criteria

We only selected papers describing the osseodensification technique with drills specific for this preparation, both used with clockwise and anticlockwise movement, with refrigeration, and with a salt solution. In the present investigation, the qualitative evaluation and meta-analysis were performed only in animal studies while no randomized clinical trial was identified by the electronic database screening. We have considered papers with statistic values of *p* < 0.005, for the choice of papers about operations on animals we only selected those following the ARRIVE guidelines. The papers excluded are those without bone compaction, whose statistic value was different from *p* < 0.005, in which there was missing information about osseodensification with suitable drills or patients submitted to it.

### 2.3. Study Selection

**Figure 1:**
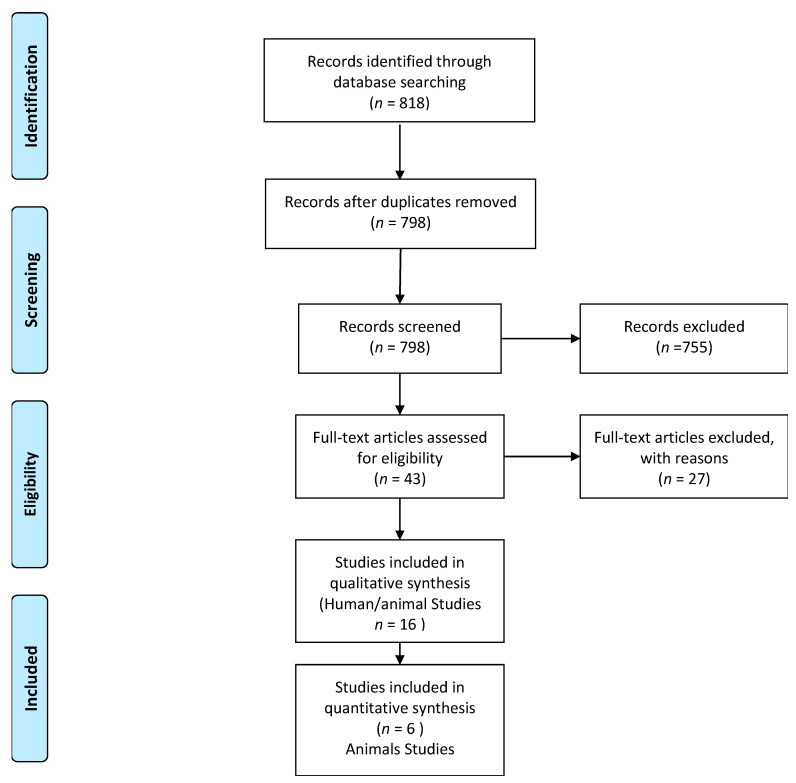
Studies screening and inclusion for qualitative analysis and meta-data evaluation processes [63].

All the included articles were full text, chosen by their title and abstract. Each one was studied independently according to the inclusion and exclusion criteria mentioned above (Figure 1). The majority of the papers were in the English language, and we only choose the ones in which the drilling technique was performed following the guidelines of the burst producer. The minimum follow-up period was set to three weeks.

### 2.4. Data Extraction

We considered useful and extract the following data from the articles we analyzed: the sample, the type of implant used in the surgery technique, the number of implants placed, the comparison of the new technique with the conventional ones, or other surgical approaches utilized in low-density bone areas, the BAFO, BIC, and IT index. We also gave importance to the follow-up period and the method of execution of the bone compaction technique.

### 2.5. Critical Appraisals

To avoid the risk of utilizing poor statistic evidence studies, we set the parameter of *p*-value < than 0.005 to consider useful an article for our review, and we use only articles that consider the BAFO, BIC, or IT index as an adequate index for the primary implant stability measurement. Moreover, we made sure all included papers describe the bone compaction technique as the guidelines describe it. We studied the sample management of each article analyzed and evaluated if they met the inclusion criteria and eliminated any possibilities of distorting result, such as systematic processes that can affect the bone quality of the subject, or the indiscriminate use of antibiotics and any drugs that can manipulate the post-surgery results.

### 2.6. Meta-Analysis Methodology and Risk of Bias Assessments

A special database (Excel, Microsoft, Redmond, WA, USA) was used for the study data collection. The meta-data analysis was performed between the clockwise and counter-clockwise procedures on iliac crest sheep model studies. The papers not conforming to the criteria were not included. The average differences were conducted for continuous variables if at least four studies were included. The evaluation was performed using the software RevMan 5.5 (The Nordic Cochrane Centre, The Cochrane Collaboration, Copenhagen, Denmark 2014). The variables considered were implant insertion torque, BIC, and BAFO histomorphometry measurements.

The risk of bias evaluation was performed in accordance with the ARRIVE guidelines for animal researches. The assessed risk of bias parameters was the ethical statement, completeness of the experimental process description, completeness of animal details (such as age, gender, weight), randomization process, selection and detection bias, population sample size determination, attrition bias, statistical evaluation, and conflict of interests. The risk of bias was defined as adequate, unclear, or inadequate. A low-risk study was determined for at least 7/10 adequate risk for each parameter. The measurement was conducted using the software RevMan 5.5.

## 3. Results

The papers selected have been entirely analyzed to reach the purposes of this study. From this analysis, the results are those reported in the following table (Table 2).

**Figure 2:**
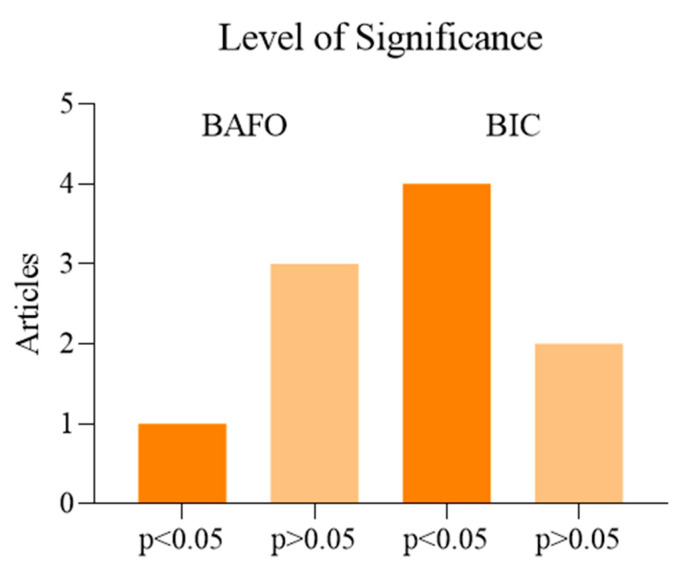
Comparison of the statistic value *p*. between the conventional technique of implant site preparation and technique with the use of drills for osseodensification. Parameters used BAFO (considered in 4 studies of 11) and BIC (considered in 6 studies of 11). For *p* < 0.05 we considered statistically valid the favorable results obtained by the osseodensification technique compared to the conventional technique.

**Figure 3:**
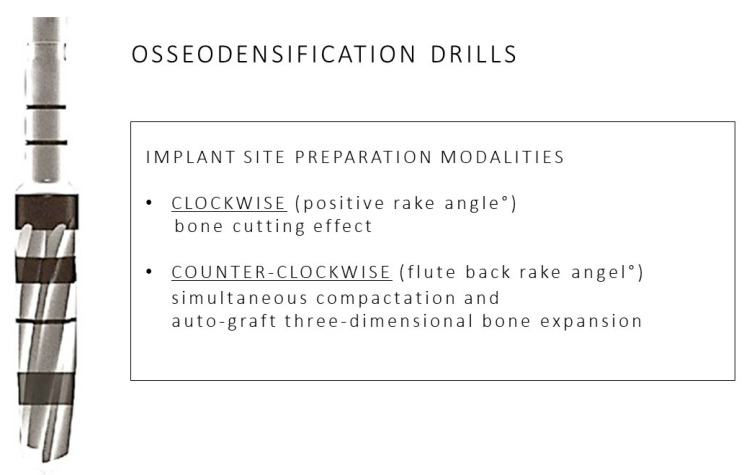
Main characteristics of the osseodensification drilling technique: details of clockwise and counterclockwise implant site preparation modalities.

**Figure 4:**
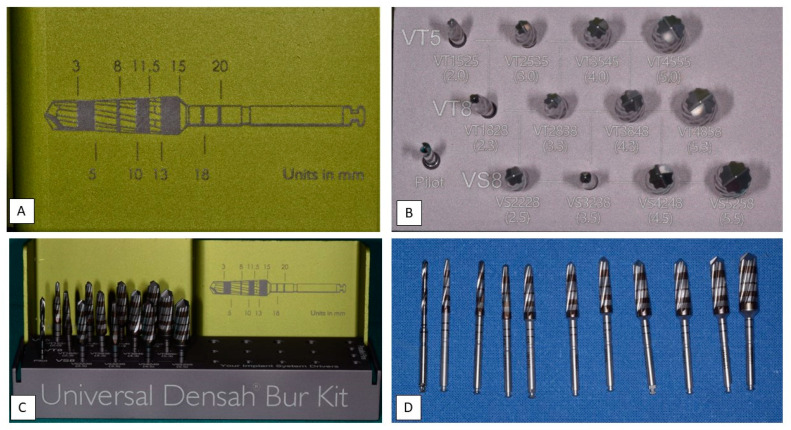
Details of the osseodensification drills system. (**A**) description of the cutters with an indication of the depth of the bone from 3.00 mm to 20 mm of the method “implant drilling with bone compaction instrumentation technique.” (**B**) Complete osseodensification Kit 13. “implant drilling with bone compaction instrumentation technique.” (**C**) Complete kit of all the cutters Versah^®^ (includes all the 13 cutters) with the method “implant drilling with bone compaction instrumentation technique.” Autoclavable kit at 137°. (**D**) Cutters in progressive order of the method “implant drilling with bone compaction instrumentation technique.”

**Figure 5:**
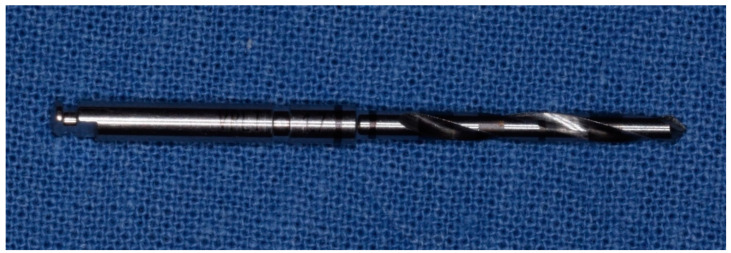
Initial drilling pilot cutter of the method “implant drilling with bone compaction instrumentation technique.”

**Figure 6:**
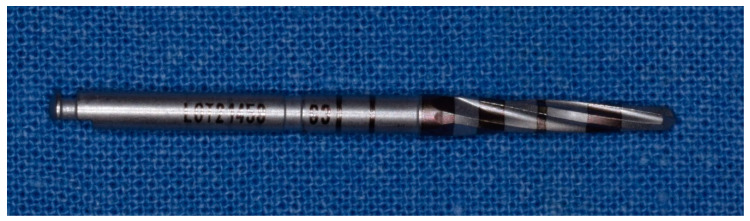
Second cutter with a diameter of 2.0 mm in the method “implant drilling with bone compaction instrumentation technique.”

**Figure 7:**
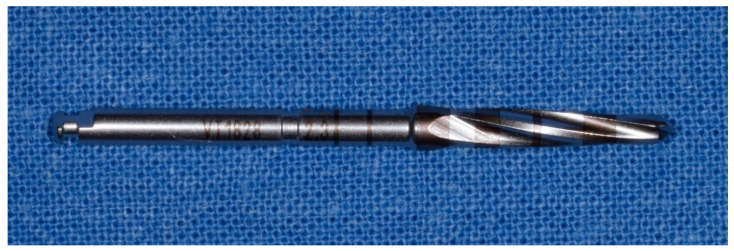
Third cutter with a diameter of 2.3 mm in the method “implant drilling with bone compaction instrumentation technique.”

**Figure 8:**
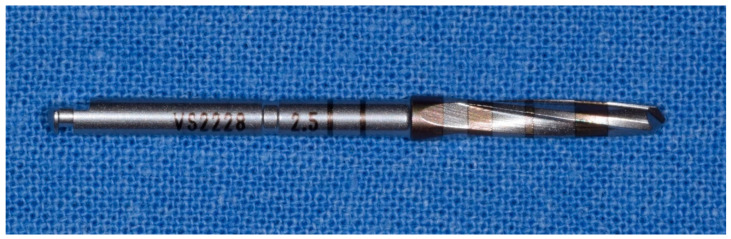
Fourth cutter with a diameter of 2.5 mm in the method “implant drilling with bone compaction instrumentation technique.”

**Figure 9:**
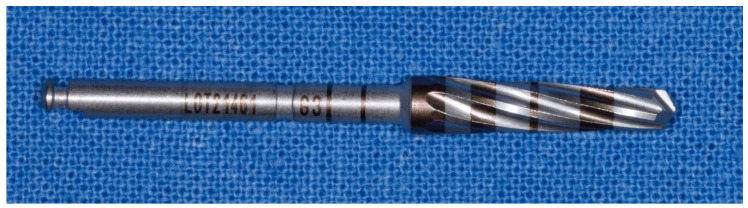
Fifth cutter with a diameter of 3.0 mm in the method “implant drilling with bone compaction instrumentation technique.”

**Figure 10:**
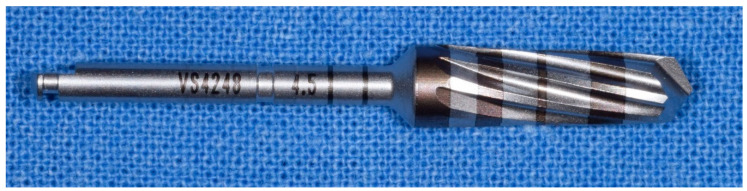
Tenth cutter with a diameter of 4.5 mm in the method “implant drilling with bone compaction instrumentation technique.”

**Figure 11:**
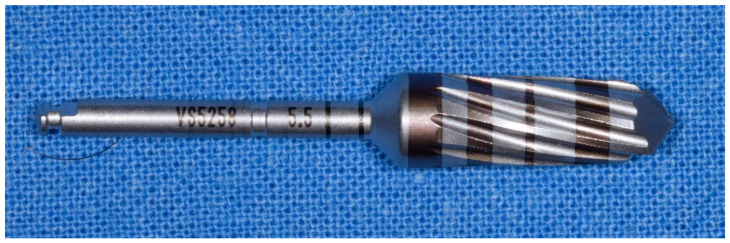
The 13th and last cutter with a diameter of 5.5 mm, method “implant drilling with bone compaction instrumentation technique.”

As resulting from the table previously described, the alveolar preparation performed with drills for osseodensification allows us to increase the surface of contact between the surface of the implant and the autologous bone of the patient [66,70,71,73,74,75]. Moreover, we may consider how the use of drills for osseodensification with anticlockwise movement (REVERSE) allows us to preserve and compact the residue bone in the immediate proximity of the implant in a more effective way than the use of clockwise movement [64,68,69,71,72,73]. We analyzed another comparison about the quantity and quality of the autologous bone maintained by the preparation with osseodensification than the Summers osteotomes, which has reported a BIC higher than 19.4% with the use of the technique with drills Versah (Densah, MI, USA) [32]. A total of eight studies analyzed was on animal subjects: six on ovine, in which we used the region of their iliac crest, two on swine (one study has used the atrophied alveolar crest, and the other one a portion of their tibia); three studies have been performed on human model (one on areas with poor bone density, one in health alveolar crest, and one in the anterior portion of the upper maxillary). The quantity of the implants placed varies in each research analyzed, i.e., 12, 18, 20, 28, 30, 36, 46, 60, 72, with several follow up 6–12 weeks [65], 3–12 weeks [35] 2 months [69], 3–6 weeks [67], 6–8 months [70], 3–6 weeks [72], 6 weeks [60], and 3 weeks [64]. The values used to compare the several techniques are BIC [62,66,70,73,75], BAFO [66,71,72,73] (Figure 2), insertion torque [67,69,70,72,74], biomechanical analysis [69], histological analysis [71,73,75], ISQ [67], and histomorphometry analysis [60]. Moreover, it is important to underline the difference in the execution of the compared techniques (Figure 3), i.e., preparation for osseodensification: pilot drill 1.5 mm, followed by the osseodensification drills Versah^®^ used with anticlockwise movement at 900–1200 rpm with irrigation [67]; conventional preparation: pilot drill 1.7 mm, followed by the drills recommended by the producers until the desired diameter (4.7 mm), technique with Summers osteotomes: pilot drill 1.7 mm, followed by the osteotomes until the compaction of the desired area, I, II, III; technique of osseodensification: pilot drill 1.7 mm, subsequent drills of diameter 2.5 mm, 3.5 mm, and 4.5 mm, with irrigation [32]; conventional preparation: pilot drill at 800–1000 rpm, followed by the drills recommended by the producer until the desired diameter, preparation for osseodensification: pilot drill with clockwise movement at 800–1500 rpm with abundant irrigation, then drills for osseodensification until the desired diameter [42]; conventional preparation: pilot drill 2 mm, drills 3.2 mm, and 3.8 mm, preparation for osseodensification with clockwise movement: pilot drill 2 mm, pilot drills 2.8 mm and 3.8 mm, preparation for osseodensification with anticlockwise movement: pilot drill 2 mm, drills 2.8 mm and 3.8 mm, and the three preparations have been performed at 1100 rpm with salt irrigation [68]; conventional preparation: pilot drill 2 mm, conventional drills 3.2 mm and 3.8 mm, preparation for osseodensification with clockwise movement: pilot drill 2 mm, drills, 2.8 mm and 3.8 mm, and the three preparations have been performed at 1100 rpm with salt irrigation [39]; conventional preparation: pilot drill 2 mm, conventional drills 2.8 mm and 3.4 mm, following the protocol Zimmer Biomet until the desired diameter, preparation for osseodensification with clockwise and anticlockwise movement: pilot drill 1.7 mm and drills 2.8 mm and 3.8 mm, the three preparations have been performed at 1100 rpm with salt irrigation [64]; preparation for osseodensification with anticlockwise movement: pilot drill followed by the drills until obtaining an alveolar site of diameter lower than the one of the implant designated of 0.5–0.8 mm, by using a speed of 800 rpm with abundant irrigation, with insertion torque of 35 Ncm [66]; conventional preparation: pilot drill 2 mm, conventional drills of 2.8 mm and 3.4 mm, preparation for osseodensification with clockwise and anticlockwise movement: pilot drill 1.7 mm performed by the drills 2.8 mm and 3.8 mm, the three preparations have been performed at 1100 rpm with salt irrigation [73]; conventional preparation: pilot drill 2 mm, conventional drills 3.2 mm and 3.8 mm, preparation for osseodensification with clockwise movement: pilot drill 2.00 mm, drills, 22.8 mm and 3.8 mm, preparation for osseodensification with anticlockwise movement: pilot drill 2.00 mm, drills 2.8 mm and 3.8 mm [68]; preparation for osseodensification: pilot drill 2 mm at 1200 rpm, drill VT1828 in REVERSE mode at 1200 rpm, drill VT 2838 in REVERSE mode at 1200 rpm, and drill VT 3848 in REVERSE mode at 1200 rpm [69] (Figure 4, Figure 5, Figure 6, Figure 7, Figure 8, Figure 9, Figure 10 and Figure 11). In the researches performed on human patients no signs of pain, suppuration, inflammation, peri-implantitis or factors in which there may result the failure of the implant surgery have been detected [67,69,72].

### Meta-Analysis and Risk of Bias Measurement

A total of four comparative articles with histomorphometry BIC and insertion torque values with clockwise and counter-clockwise procedures were included. The experimental outcomes were classified according to a minimum follow-up period of three weeks [66,70,74,75].

A total of five studies were included according to histomorphometry BAFO for a comparative evaluation between clockwise and counter-clockwise procedures [66,68,70,74,75].

**Figure 12:**
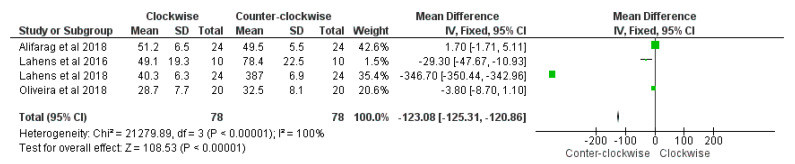
Forest plot of comparison of BIC percentage, of the clockwise procedure (right) and counter-clockwise procedure (left).

The meta-analysis procedure demonstrated a significantly higher BIC percentage between the counter-clockwise group compared to the clockwise group was present (overall effect: *p* < 0.01; Z: 108.53; heterogeneity: *p* < 0.01; χ2: 21279.89, df:3; I^2^: 100%) (Figure 12).

**Figure 13:**
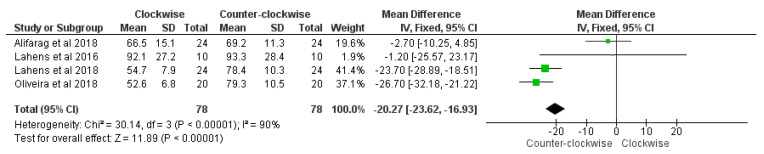
Forest plot of comparison of insertion torque, of the clockwise procedure (right) and counter-clockwise procedure (left).

A significantly higher insertion torque between the counter-clockwise group compared to the clockwise group was highlighted (overall effect: *p* < 0.01; Z: 11.89; heterogeneity: *p* < 0.01; χ2: 30.14, df:3; I^2^: 90%) (Figure 13).

**Figure 14:**
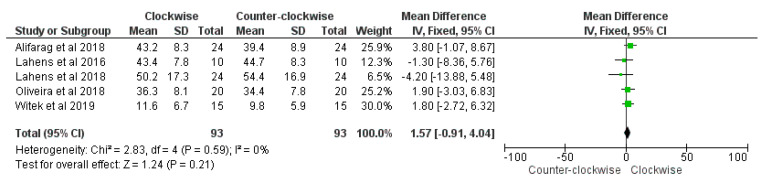
Forest plot of comparison of insertion torque, of the BAFO (right) and counter-clockwise procedure (left).

No significant difference of histomorphometry BAFO percentage between the counter-clockwise group compared to the clockwise group was reported (overall effect: *p* = 0.21; Z: 1.24; heterogeneity: *p* = 0.59; χ2: 2.83, df:4; I^2^: 0%) (Figure 14).

**Figure 15:**
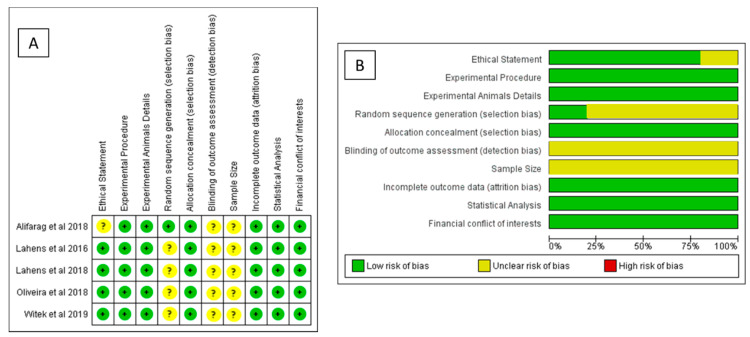
Risk of bias measurement: (**A**) summary of risk of bias for each included study (left) and (**B**) summary of each risk of bias item presented as percentages across all included studies (right).

The risk of bias measurement was conducted on all studies included for the meta-analysis and summarized in Figure 15A,B, where a total of five studies on animals showed a low risk of bias [66,68,70,74,75].

The included papers showed the same animal model design, experimental site and defect, methods, and comparable measurements.

## 4. Discussion

The present review of the scientific literature has the purpose to study the validity of the use of the technique of preparation of osseodensification as a useful technique for implant surgery. The analyzed studies are contradictory; in some, there are solid results to confirm this technique, supported by some statistically relevant values [60,64,65,66,68,71,72,73], but other studies reported no data that show the scientific difference in relation to the conventional technique [69,70,71]. The conventional osteotomy is considered a subtractive surgery [54,74] because it removes autologous bone from the insertion site of the implant, while the technique for the osseodensification compacts it and models in favor of the implanted graft [64,75]. It is possible to notice that most part of the analyzed studies confirms the osseodensification for what concerns the maintaining of the quality and quantity of autologous bone, which will influence the result of the implant surgery in a notable way [76] because it ensures the primary stability of the implant placed [62]. It has been hard to compare the journals because they differ according to the method of study, used materials, subjects selected for the experimentation, indicators of assessment of the results, follow-up, and other information. Nevertheless, this analysis has given us a global vision of the results obtained by the osseodensification technique and its possible use. In the literature, we can find sporadic case reports about osseodensification [77,78,79], and also in these cases, there is evidence about the efficacy of this technique [77]; instead, positive results have been observed in studies that compare the preparation technique for osseodensification and the conventional technique of implant preparation in blocks of polyurethane in several densities in which the innovating technique has been shown advantages especially in areas where the obtaining of good primary stability would have been harder [11]. Several alveolar preparation techniques have been described to increase the interface of the implant with surrounding bone [80] in order to improve the primary stability and the osseointegration outcomes. The interface implant–bone matters in terms of primary stability, decreasing the chances of implant micromovements, which is one of the main causes of implant loss [52,81,82,83,84,85,86], so the research on methods to enhance this value shall be a priority in the foreseeable future. The osseodensification technique might find application in various fields of surgeries, such as orthopedic surgery, where screw failure remains a severe complication that needs to be overcome [81] with further studies and trials. For the literature issued until now about the osseodensification, including above all studies on animals, few cases, analyzed serially or individually, it is harder to assess the efficacy of this technique about the real increase of primary stability. In the present investigation, only the animal studies on sheep were considered for quantitative analysis according to the similitudes of the study model design, methodological analysis, and follow-up with a sufficient quantity of papers selected. The other human and animal study models did not present the requirements for a meta-analysis evaluation. The non-randomized human studies included seems to confirm the effectiveness of the technique for implant osteotomy in poor bone density reported in animal models. Moreover, the evidences of the present investigation highlighted a difference of efficiency of the two counter-clockwise and clockwise protocol for osseodensification drills in terms of insertion torque and BIC% after three weeks of healing in low-density bone. Clinically, the counter-clockwise drilling technique is able to determine a significant increase of local bone density with a simultaneous bone compaction and three-dimensional autografted expansion [70,73,75] and to promote the primary stability occurring the dental implant positioning [71,73]. In the literature, an insertion torque value of ≃35 Ncm is considered a fundamental clinical condition of optimal primary stability and the long-term predictability of dental implant rehabilitation, that could be clinically affected by poor bone density jaws anatomies, such as the posterior maxilla [82,83]. Moreover, no difference of bone area fraction occupancy % (BAFO) were detected between the surgical drilling technique after the healing period. We need in vivo studies on animals and humans with important follow up in order to provide solid clinical recommendations. Several studies have proved how osteotomes technique can be a valid solution to obtain an improvement in primary stability while preserving bone tissue [59,60], and osseodensification has the same aims with an innovative approach related to recent technologies. The analyzed papers in this bibliographic review detect no conflict of interest [37,62,64,65,66,71,72] except for the authors S. Huwais, as inventor of the drills Densah^®^ and pioneer of the osseodensification [67,84], P. Trisi, who used Cortex implants for his study, a company of which he is consultant [69], and F.B. Slete and P. Olin, both with a minimal financial interest in the company Versah^®^, LLC. [60].

## 5. Conclusions

Literature is lacking in papers concerning the osseodensification and limited to studies on animals and clinical cases with short-term follow up, which do not allow us to perform an objective assessment of the advantages of the technique treated; one of the causes is surely the innovativeness of the drills for osseodensification, which still today are not part of the standard implant clinical practice. This technique seems to be promising in the case in which the autologous bone is poor in quality (i.e., cases in which the missing dental element lasted up to provoke the atrophy of the autologous bone of the patient, or very hard areas for the implant primary stability by respecting of the noble anatomic areas), as it “compacts” and “respects” the bone that is directly adjacent to the graft site of the implant. If we consider the techniques with drills for osseodensification from a practical point of view, we would notice the need for suitable training courses for the use of these tools because they are an important part of the practice and need highly skilled clinicians and certain confidence (in order to reach the effect of osseodensification, the drills give a feeling of “hammering” on the surgical handle, which would make it complicated to maintain the path of work designed in hands with poor experience). Further studies would turn to the use of drills in cases in which a maxillary sinus lift would be necessary because, due to their potential in considering the tissue that would face the necessity of this operation, they can prove beneficial and the study of the efficacy of this technique in this direction would result in very notable clinical advantages in the modern implantology by detecting the cases in which this is the choice to make. Despite the results reached about the osseodensification technique with specific drills are modest and “immature,” they need to be read very carefully. The demand should increase together with the setting of new studies on humans and animals in vivo with long-term follow-up to include the technique of bone compaction in the implant everyday practice.

## Figures and Tables

**Table 1:** PICO (population, intervention, comparison, outcome) questions explication.

Population\Patients	Intervention	Comparison	Outcomes
Patient group of interest?	What is the main intervention you wish to consider?	Is there an alternative intervention to compare?	What is the clinical outcome?
Patients that need oral rehabilitation with dental implant surgery in low-density bone areas	Implant positioning with the bone compaction technique	Conventional implant Site preparation	Can this technique provide optimum primary implant stability?

**Table 2:** Comparison of the papers analyzed according to the choice of the sample of these studies, the techniques used, the model and type of implants, the results obtained. BAFO, bone area fraction occupancy; BIC, bone-implant contact; IT, insertion torque; OD, osseodensification technique through alveolar preparation, OSO, osseodensification technique through alveolar preparation with drills used in a clockwise direction; OAO, technique of osseodensification through alveolar preparation used in an anticlockwise direction; C, conventional technique of alveolar preparation; CS, technique that uses Summers osteotomes.

Authors	Study Model	Techniques	Implants Type	N implants	BAFO	BIC	IT
Alifarag et coll. 2018 [64]	Ovine iliac crest	Conventional; osseodensification preparation (clockwise and anticlockwise)	Tapered screw vent Trabecular metal (Zimmer)	36 (18 TSV; 18 TM)		OAO > C *p* = 0.037 OSO > C *p*= 0.005 OAO\OSO *p* > 0.05	
Hindi et coll. 2020 [65]	Humans	osseodensification preparation	-Diameter 4.1 mm (26;56.2%) 3.5 mm (20;43.8%) -Length 10 mm (21;45.6%) 12 mm (19;41.3%) 8 mm (6;13.1%)	46			>35 Ncm 35 implants (76.1%) =35 Ncm 11 implants (23.9%)
Witek et coll. 2019 [66]	Ovine iliac crest	Conventional; osseodensification preparation (clockwise and anticlockwise)	TM (Zimmer) 3.7 mm diameter 10 mm length		OAO > C *p* = 0.036	OD > C *p* > 0.05	
Koutouzis et coll. 2019 [67]	Humans	osseodensification preparation	TSV (Zimmer)	28			Immediate post-operative +\− 61.3 Ncm, after 3 and 6 weeks respectively +/−56.6 Ncm and +/−59.8
Lahens et coll. 2018 [68]	Ovine iliac crest	Conventional; osseodensification preparation (clockwise and anticlockwise)		72 implants, 36 treated with acid; 36 treated mechanically		OSO > C (*p* = 0.024) OAO > C (*p* = 0.006)	OSO + OAO > C (*p* < 0.001)
Trisi et coll. 2016 [69]	Ovine iliac crest	Conventional; osseodensification preparation	Dynamic Implant (Cortex)	−10 implants 3.8 mm diameter; 10 mm length −10 implants 5 mm diameter 10 mm length		C = 46.19% +/− 3.98%; OD = 49.58% +/− 3.19%	
Sultana et coll. 2020 [70]	Humans anterior maxilla	Conventional; osseodensification preparation	Tuareg S (Adin)	20 Several diameters and longitudes			OD = immediate post operation 65.6; after 6 months 66 OD = 57.6 immediate post operation; after 6 months 64.8 OD\C = *p* > 0.05
Tian et coll. 2019 [71]	Swine, mandibular crest	Summers osteotomes; osseodensification preparation		12 4 mm diameter 13 mm length	OD > C *p* = 0.198	C = 31.4% OD = 62.5% OD > C *p*= 0.018	
Slete et coll. 2018 [60]	Swine tibia	Conventional + Summers osteotomes; osseodensification preparation	TSV (Zimmer)	18 4.7 mm diameter 13 mm diameter		OD = 60.3% CS = 40.7% C = 16%	
Oliveira et coll. 2018 [72]	Ovine iliac crest	Conventional; osseodensification preparation (clockwise and anticlockwise)		60, conical, 4 mm diameters 10 mm length (30 with surface treated with acidifiers, 30 with only mechanic treatment)	OD > C = *p* = 0.330	OAO = +/−31% OSO = +/−28% C = +/−24% OD > C = *p* = 0.148	C = 10 Ncm OSO = 53 Ncm OAO = 78 Ncm OAO > OSO > C = *p* < 0.005
Lahens et coll. 2016 [73]	Ovine	osseodensification preparation (clockwise and anticlockwise)	Axis Tag	30 4.2 mm diameter 10 mm length	OD > C = *p* = 0.22	C = 50% OSO = 60% OAO = 70% OD\C = *p* < 0.05	C = 25 Ncm OSO = quasi 100 Ncm OAO = quasi 100 Ncm OD\C = *p* < 0.001

## Data Availability

All experimental data to support the findings of this study are available contacting the corresponding author upon request.
